# Altered [^99m^Tc]Tc-MDP biodistribution from neutron activation sourced ^99^Mo

**DOI:** 10.1007/s10967-018-5826-0

**Published:** 2018-03-29

**Authors:** Sandor Demeter, Roman Szweda, Judy Patterson, Marine Grigoryan

**Affiliations:** 1 Faculty of Health Sciences, College of Medicine, University of Manitoba, Winnipeg, Canada; 2 Section of Nuclear Medicine, Winnipeg Health Sciences Centre, Winnipeg, Canada; 3 Radiopharmaceuticals Research Group, Health Sciences Centre, Winnipeg, Canada

**Keywords:** Biodistribution, Radiopharmaceutical, Quality control, Neutron activation, ^99^Mo, Pertechnetate

## Abstract

Given potential worldwide shortages of fission sourced ^99^Mo/^99m^Tc medical isotopes there is increasing interest in alternate production strategies. A neutron activated ^99^Mo source was utilized in a single center phase III open label study comparing ^99m^Tc, as ^99m^Tc Methylene Diphosphonate ([^99m^Tc]Tc-MDP), obtained from solvent generator separation of neutron activation produced ^99^Mo, versus nuclear reactor produced ^99^Mo (e.g., fission sourced) in oncology patients for which an [^99m^Tc]Tc-MDP bone scan would normally have been indicated. Despite the investigational [^99m^Tc]Tc-MDP passing all standard, and above standard of care, quality assurance tests, which would normally be sufficient to allow human administration, there was altered biodistribution which could lead to erroneous clinical interpretation. The cause of the altered biodistribution remains unknown and requires further research.

## Introduction

The majority of diagnostic nuclear medicine studies utilize ^99m^Tc as the base medical isotope. Until very recently over 70% of the world’s supply of ^99m^Tc’s parent isotope, ^99^Mo, was produced as a fission product in two high enriched uranium (HEU) reactors, i.e., the National Research Universal (NRU) reactor at Chalk River, Canada and the high flux reactor (HFL) in the Netherlands [1, 2].

Three major factors have catalyzed a search for alternate sources of ^99^Mo/^99m^Tc.

First, both the NRU and HFL reactors are over five decades old and both are nearing, and some would say beyond, their life expectancy. Second, in 2009/10 the world experienced a critical shortage of ^99^Mo/^99m^Tc due to an unplanned shut down of the NRU reactor at the same time as planned maintenance down time of the HFL. This was a wakeup call to the world that the existing global production chain could not tolerate such perturbations in supply. Finally, and primarily for nuclear security reasons, the US Department of Energy’s National Nuclear Security Administration has mandated a transition from HEU to low enriched uranium (LEU) ^99^Mo/^99m^Tc production [1–7].

Alternate ^99^Mo/^99m^Tc sources include: low enriched uranium fission products, neutron activation (i.e., ^98^Mo(n, γ)^99^Mo), linear accelerator (e.g., ^100^Mo(**γ**, n)^99^Mo) and cyclotron (e.g., ^100^Mo(p, 2n)^99m^Tc) [8–10].

Research was conducted by the prairie isotope production enterprise (PIPE), in Winnipeg, Manitoba, Canada demonstrating proof of principle that neutron activated or LINAC produced ^99^Mo could be utilized to extract [^99m^Tc]NaTcO_4_ via liquid–liquid methyl ethyl ketone (MEK) extraction [11]. PIPE then conducted further research to demonstrate commercial viability relative to producing sufficient ^99^Mo/^99m^Tc to meet clinical demands. This later research was to dovetail with a clinical trial.

This paper focuses on the early unexpected results of the clinical trial.

## Experimental

### Radiochemistry

Procedures for the production of ^99^Mo by nuclear reactor-irradiation at the Missouri University research reactor (MURR, Columbia, MO) and [^99m^Tc]NaTcO_4_ solvent extraction have been previously described [12, 13].

### Quality control

The emphasis in this section will focus on the products used in the human clinical trial. For a detailed analysis of ^99^Mo and ^99m^Tc quality assurance between MURR and commercial generator sources readers are referred to the article by Grigoryan et al. [11].

### Methods

#### QC of [^99m^Tc]NaTcO_4_ (Pert) eluate for the preparation of The [^99m^Tc]Tc-MDP

The [^99m^Tc]NaTcO_4_ (Pert) eluate was analyzed against United States Pharmacopeia (USP) and additional specifications [14].

### Radiolabeling of MDP

[^99m^Tc]Tc-MDP was prepared from commercial MDP kits [Edmonton Radiopharmaceutical (15)] by adding 3–12 GBq of freshly eluted Pert to the kit vial. MDP kits were radiolabeled following package insert instruction, and QC performed after reconstitution. A total of 21 [^99m^Tc]Tc-MDP samples were prepared from 6 different batches of neutron activation sourced sodium molybdate and analyzed for animal (mouse) biodistribution. Four separate batches of radiolabeled MDP were used for the human clinical study.

Radiochemical purity of labeled radiopharmaceutical was evaluated using instant thin layer chromatography (iTLC) strips from BIODEX Medical System Inc. (models 150-001 and 1500-005) with Acetone and distilled water as mobile phases. The pH of the tagged radiopharmaceutical was determined using the pH indicator test paper 4.0–7.0 (Merk) and validated with pH meter (Orion, Thermo Scientific).

### Sterility

BacT/ALERT^®^ Microbial Detection System and culture bottles (BIOMEREUX INC.) were used in qualitative procedures for enhanced recovery and detection of aerobic and facultative microorganisms (bacteria and fungi) in the fresh eluent and [^99m^Tc]Tc-MDP.

### Pyrogen content

Bacterial endotoxins were quantified by chromogenic methods using point-of-use test system Endosafe^®^-PTS (Charles River Laboratory). Samples were diluted 50 times.

### Biodistribution study

[^99m^Tc]Tc-MDP was administered intravenously (70–100 MBq in 0.2 mL) in CD1 mice (*n* = 24). The animals were sacrificed at 10 min, and 2 h post injection and organ tissues were removed, washed and weighted. Radioactivity was determined in a gamma counter (HIDEX AMG Automatic Gamma Counter. The results were expressed as a percent of administered dose/organ.

## Results

### QC of neutron activation sourced [^99m^Tc]NaTcO_4_ eluate (MURR Pert) for the preparation of [^99m^Tc]Tc-MDP

Overall 100 extractions of MURR sourced [^99m^Tc]NaTcO_4_ (Pert) from 15 batches of sodium molybdate were analyzed.

Results for MURR sourced Pert used for the preparation of MDP samples utilized in clinical trials are summarized in Table 1.

**Table 1:** QC results for MURR [^99m^Tc]NaTcO_4_ (Pert) used in radiolabeling of MDP for clinical trial (*n* = 4 study participants)

Tests	Results				Specifications
Lot number^a^	M13-16-004	M13-16-007	M13-16-008	M14-16-002	
Radiochemical purity					
Radio-iTLC	99.55%	99.5%	99.55%	99.37%	> 90%
Moly breakthrough	0.00	0.00	0.00	0.00	< 0.15
Chemical purity					
Aluminum test	Pass	Pass	Pass	Pass	< 10 µg
Iodoform	Pass	Pass	Pass	Pass	< 0.1%(v/v)
Sterility	Pass	Pass	Pass	Pass	pass/fail
Pyrogenicity	Pass	Pass	Pass	Pass	pass/fail
pH	6.34	5.39	5.47	6.05	4.5-7.5

^a^Perts used for radiolabeling MDP for patients

All radio-iTLC results were above the passing 90% limit, with the majority being well above 95%. Aside from two extractions which had pH values above passing specifications (Pert from these extractions were not used in the clinical trial), all others were within the USP range of 4.5–7.5. A trend of decreasing pH with subsequent extractions was observed and the same trend was observed in pertechnetate obtained from the commercial Ultra-Technikov^TM^ V4 generator (Mallinckrodt Pharmaceuticals). All MURR sourced Pert used for the clinical trial passed all quality assurance tests.

^99^Mo breakthrough results for the MURR Pert were all within the passing specification of < 0.15 kBq ^99^Mo/MBq. Moly breakthrough was typically higher, but always within specification, for the first extraction of the generator and then decreases through subsequent extractions.

**Fig. 1:**
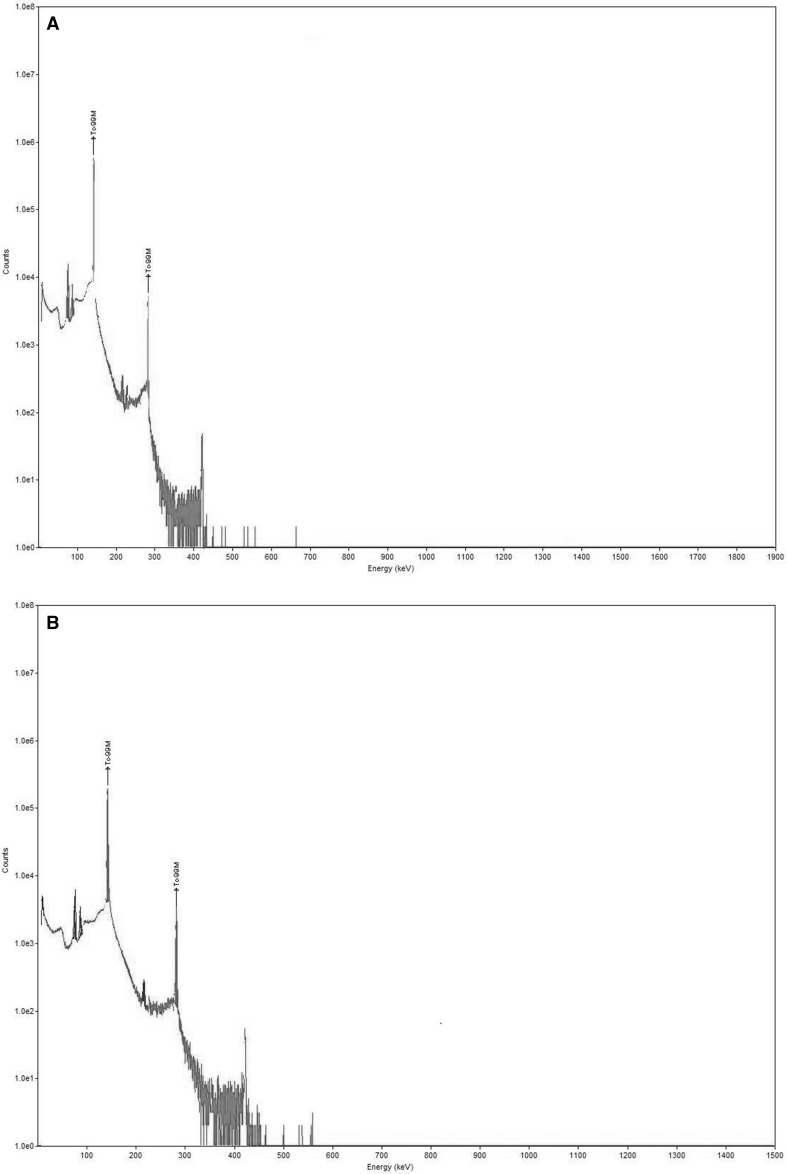
Gamma-spectra of sodium pertechnetate extracted from: **a** A commercial Ultra-Technikov^TM^ V4 generator (Mallinckrodt Pharmaceuticals) used in the Radiopharmacy department of Health Sciences. **b** The primary source ^99^MoO_4_^2−^ solution produced by dissolving nuclear reactor-irradiated sintered ^99^Mo discs (MURR)

Germanium spectra of the pertechnetate obtained from commercial generator, Fig. 1a and MURR Pert, Fig. 1b, and show no significant observable differences. Both show main peak at 140 keV and summation peak of 280 keV, which corresponds to ^99m^Tc, with no other significant peaks (both samples immediately after extraction, i.e. 0 h).

All extractions of pertechnetate passed sterility and pyrogenicity tests.

### Radiolabeling of MDP

A total of 21 preparations of MDP using MURR Pert were made which include the 4 radiolabeled products used in the clinical trial (Table 2).

**Table 2:** QC results for radiolabeled MDP used in clinical trials

Lot#	pH (spec. 6.0–6.5)	Radio-iTLC (spec. > 90%)		Sterility
		Acetone (%)	Water (%)	
MURR13-MDP1	6.29	98.49	99.17	Pass
MURR13-MDP2	6.16	99.55	98.90	Pass
MURR13-MDP3	6.19	99.27	98.32	Pass
MURR14-MDP1	6.30	95.60	98.60	Pass

Passing specification for [^99m^Tc]Tc-MDP radio-iTLC is ≥ 90%. The majority of the acetone and water radio-iTLC results were > 95% and were similar to results for radiolabeled MDP when pertechnetate from a commercial generator was used. All radiolabeled MDPs had the pH values within the passing specifications of 6–6.5.

### Sterility and pyrogenicity

Each extraction of MURR Pert passed tests for sterility and pyrogenicity and all radiolabeled MDPs passed sterility tests.

### Animal biodistribution study

[^99m^Tc]Tc-MDP at (standard dose of 70–100 MBq) was administered by intravenous tail injection into 24 CD1 mice. Institutional ethical approval^1^ was obtained for the animal study.

**Fig. 2:**
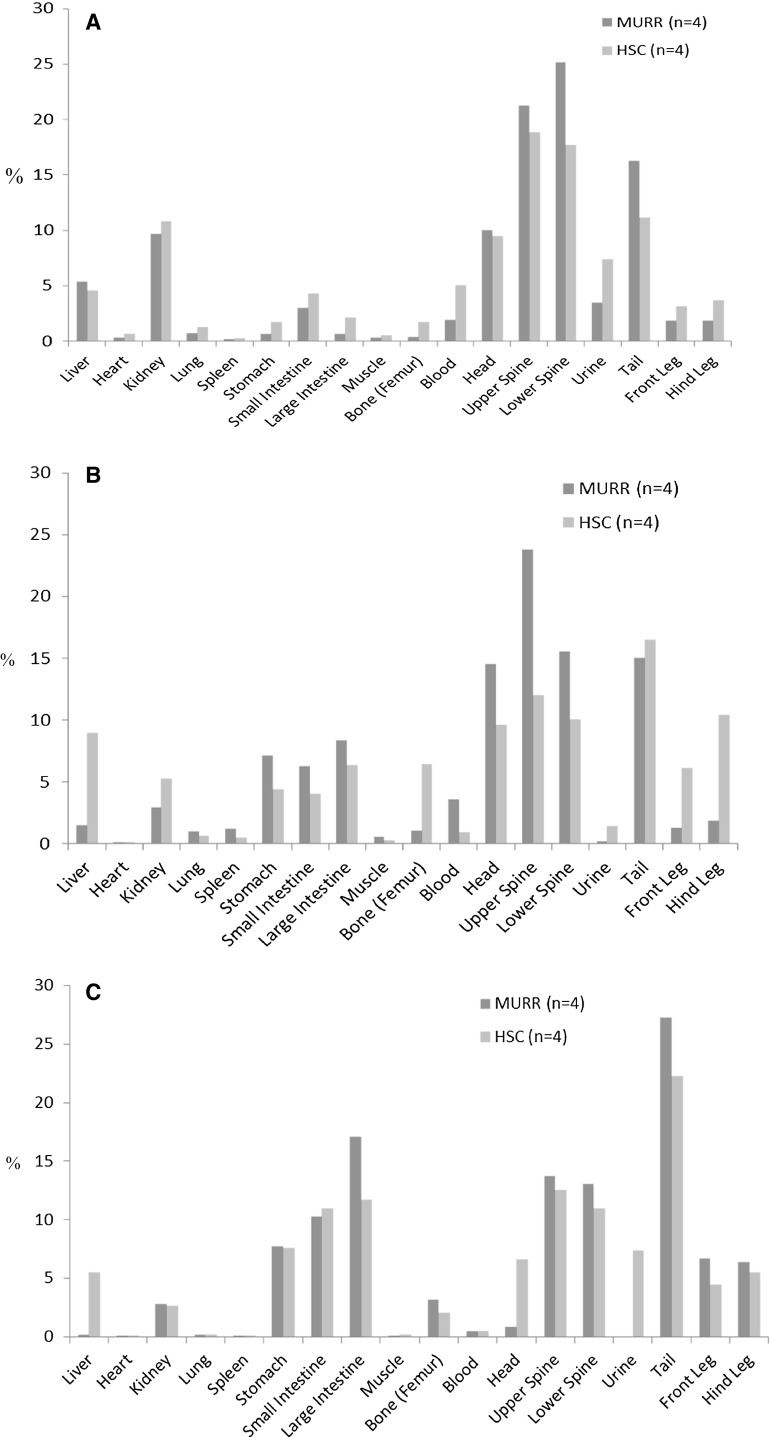
Biodistribution (%ID/g) of MURR [^99m^Tc]Tc-MDP and commercial [^99m^Tc]Tc-MDP at various time points: **a** 10 min; **b** 2 h; **c** 4 h. Sample size for each timpoint were *n* = 4 for injection. The timepoints shown represent the time after administration of the dose

Animals were sacrificed at 10 min, 2, and 4 h post injection and dissected to measure the percent of total activity in each organ using a HIDEX Gamma counter. There were four mice (n = 4) done at each time point for both MURR [^99m^Tc]Tc-MDP and for commercial [^99m^Tc]Tc-MDP as illustrated in Fig. 2a, b and c.

Biodistribution of the MDP is very similar between the two sets of radiopharmaceuticals (MURR and commerical or HSC). Both show a gradual uptake of MDP into the GI tract as time progresses; however with a relative trend of increasing large bowel uptake of MDP for the MURR MDP injected mice.

**Fig. 3:**
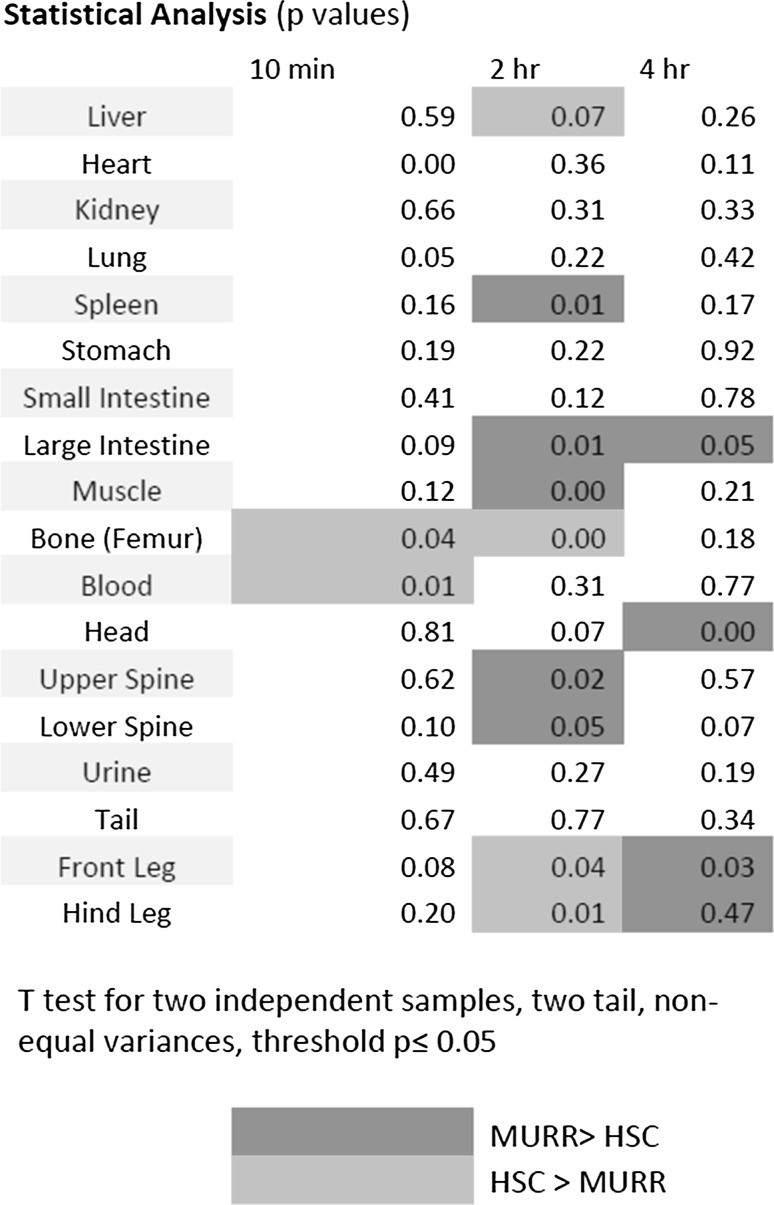
Two tail Students *t* test for independence results comparing organ/tissue uptake at 10 min and 2 and 4 h after mouse tail vein injection (*n* = 4 mice for each timepoint)

A two tail Students *t* test for independence was used to compare organ/tissue uptake at 10 min and 2 and 4 h after tail vein injection. Significant differences, at the *p* ≤ 0.05 level, were generally scattered and inconsistent. Results are presented in Fig. 3.

The MURR MDP had significantly decreased femur uptake at 10 min and 2 h but not at 4 h. MURR MDP had significantly increased spine uptake at 2 h but not at the other time points. Relative to the human imaging findings it is notable that the MURR product had significantly increased relative activity in the large intestine at both 2 and 4 h.

### Clinical trial

The trial was a prospective single center phase III open label study comparing ^99m^Tc, as ^99m^Tc Methylene Diphosphonate ([^99m^Tc]Tc-MDP), obtained from solvent generator separation of neutron activation produced ^99^Mo, versus nuclear reactor produced ^99^Mo (e.g., fission sourced) in oncology patients for which an [^99m^Tc]Tc-MDP bone scan would normally have been indicated. The study participants acted as their own controls (i.e., standard of care bone scan first followed by MURR [^99m^Tc]Tc-MDP in same patient 3–28 days later). The clinical study was approved by Health Canada and the institutional Research Ethics Board and is listed as “terminated” in https://clinicaltrials.gov/ under the trial registration number NCT03002454.

### Inclusion criteria

1. Male or female patient oncology out-patients in which a standard of care [^99m^Tc]Tc-MDP bone scan has been already been obtained.
2. If female of child-bearing potential is outside of the window of 10 days since the last menstrual period, a negative serum or urine pregnancy test is required.
3. Age greater than or equal to 18 years.
4. Able and willing to follow instructions and comply with the protocol.
5. Deemed competent to provide written informed consent prior to participation in this study.

### Exclusion criteria

1. Nursing or pregnant females.
2. Age less than 18 years.
3. No written consent provided.
4. Interval since a prior bone scan less than 3 days or greater than 3 weeks.
5. Identified interval event which could influence/change bone scan uptake (e.g., skeletal trauma, orthopedic surgery, bone infection, or interval therapy (i.e., radiation therapy, non-maintenance chemotherapy).
6. Initial bone scan of suboptimal diagnostic quality relative to non-radiopharmaceutical factors such as, partially interstitial injection or alternated image acquisition parameters (e.g., off peak acquisition).
7. Patients who exceed the safe weight limit of the scanner bed.
8. Patients who have experienced an adverse reaction to fission sourced [^99m^Tc]Tc-MDP.
9. Patients who are deemed not be able to tolerate the imaging procedure (e.g., cannot lie still for the duration of image acquisition).

Up to 50 patients were to be enrolled. (Sample size parameters included: anticipated lesion prevalence − 30%, [^99m^Tc]Tc-MDP sensitivity 90%, powered to detect a Kappa of 0.7 for visual equivalence between the set of images [15, 16]).

A set of experienced Canadian Royal College of Physicians and Surgeons certified Nuclear Medicine physicians were to be presented with paired anterior and posterior whole body planar images labeled “A” and “B” representing the neutron activated and commercial generator sourced ^99^Mo/^99m^Tc (randomly assigned to “A” or “B”). The readers were to be presented only with the image data sets and were to be blinded as to the order of the paired images.

### Imaging protocol

Administered ^99m^Tc Methylene Diphosphonate ([^99m^Tc]Tc-MDP) doses were 750 MBq (± 10%). Imaging parameters were the site’s standard dual head whole body bone scan settings which were aligned between the standard of care and the investigational agent bone scans (e.g., specific gamma camera, time between injection and imaging acquisition settings). Two patients were imaged on a Siemens Symbia T6 and two on a GE Hawkeye Infinia systems.

## Results

The trial was terminated after the first 4 patients were enrolled due to gross altered biodistribution between the two sets of images. This was noted by the study principal investigator when the image data sets were being prepared for the expert readers. These patients were recruited in a 2 week period and included the investigational agent from two separate neutron activated ^99^Mo shipments. There were two females and two males recruited with an average age of 60.5 and pathologies included: prostate cancer (*n* = 2), breast cancer (*n* = 1) and uterine cancer (*n* = 1).

**Fig. 4:**
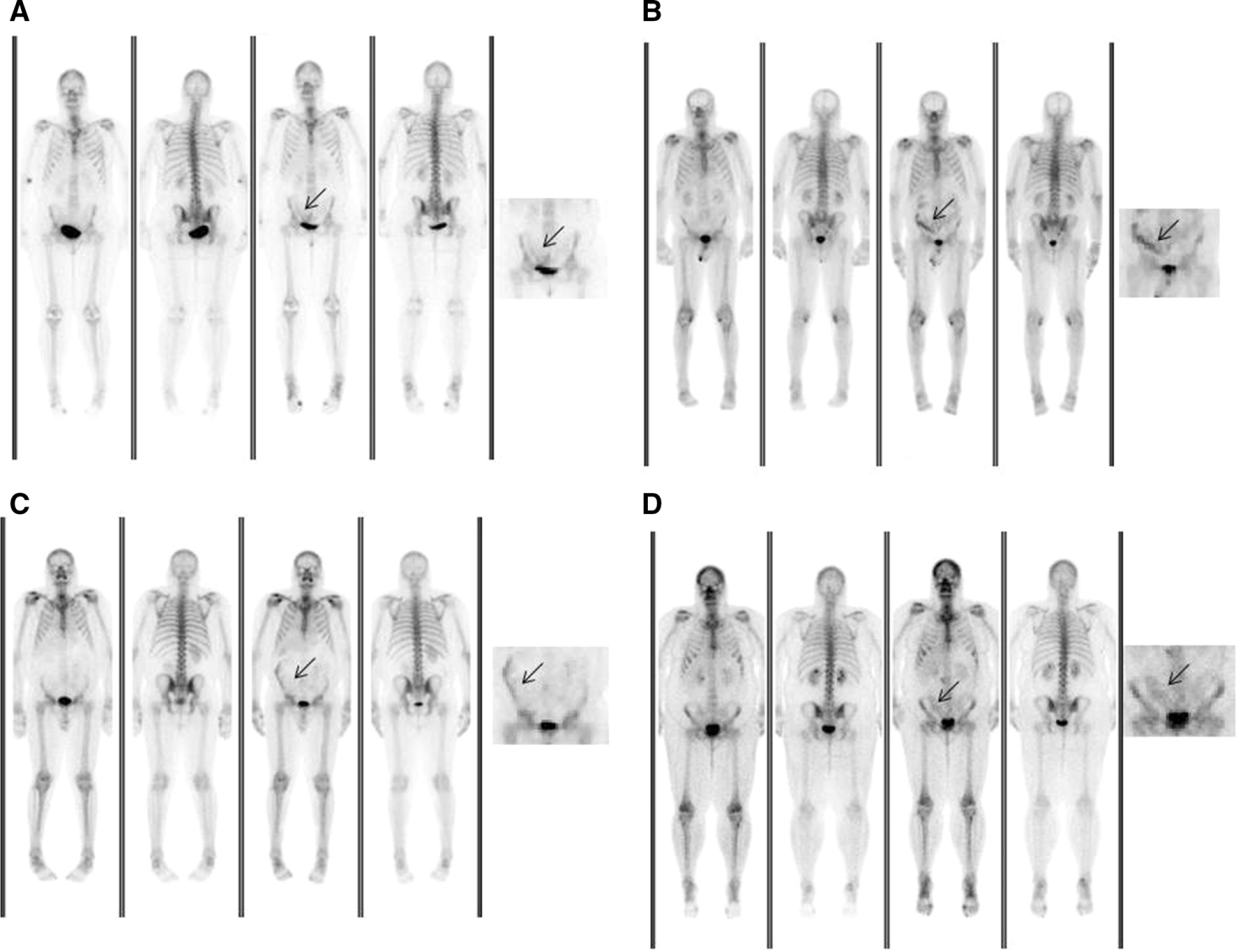
**a**–**d** Whole body bone scans of patients enrolled in the study. The left panel for each patient utilized commercial [^99m^Tc]Tc-MDP and the right panel utilized MURR [^99m^Tc]Tc-MDP. An enlarged image of the pelvis from the right panel better illustrating the altered biodistribution is provided on the right of each image set

Figure 4a–d, illustrates consistent altered biodistribution of “bowel like” uptake (arrow) in the right lower quadrant for the four participants (standard of care bone scan on the left and investigational agent on the right).

After a chart review there were no protocol breaches for these participants, no significant interval interventions, no acute symptomatic episodes, and no known interfering medications to account for the findings.

To reiterate, and add to, the previous section, the MURR [^99m^Tc]Tc-MDP passed all quality control parameters including some beyond standard of care testing (e.g., radio-iTLC (Pert and MDP), pH (Pert and MDP), Germanium spectroscopy (Pert), ^99^Mo breakthrough, and mass spectroscopy (Pert).

## Discussion

Elution of generator and radiolabeling of commercial MDP was performed by experienced technologists according to existing SOP’s. Proper handling procedures were proven many times during regular Radiopharmacy production. Intervals between Pert elution were less than 24 h to minimize formation of ^99^Tc (i.e., cold pert) that can compete with ^99m^Tc for stannous reduction and chelation reactions. All product used in the clinical trial were prepared as specified in the commercial MDP kit monograph.

Radiation can interact with water molecules forming ions, free radicals and peroxides particularly at higher activities and is well described [17–19]. Peroxides and hydroxyl free radicals are capable of oxidizing stannous ion and interact with technetium complexes to cause decomposition and oxidation of reduced technetium resulting in production of free Pert, noting that there was no evidence of free Pert on the study participants images. Peroxides tend to build up over time and may be present in the eluate from a generator with prolonged in-growth time, or Pert aged for several hours since elution. However, ascorbic acid that is present in the MDP kit should substantially reduce radiolytic decomposition.

Galea et al. [20] published on animal [^99m^Tc]Tc-MDP and Tetrofosmin imaging using ^99m^Tc derived from either a commercial ^99^Mo generator or a linear accelerator (LINAC) source (i.e., 100Mo (*γ,* n) ^99^Mo). No abnormal bowel uptake was noted for the LINAC sourced [^99m^Tc]Tc-MDP although there was altered renal uptake in the non-buffered versus the buffered versions thought to be related to gluconate impurities.

This the first known publication demsontrating altered biostribution from neutron activated sourced [^99m^Tc]Tc-MDP in humans.

## Conclusion

This study illustrates the importance of being vigilant and managing unexpected findings when conducting a clinical trial. Despite the investigational agent passing all standard, and above standard of care, quality assurance tests, which would normally be sufficient to allow human administration, there was altered biodistribution which could lead to erroneous clinical interpretation. The cause of the altered biodistribution remains unknown and requires further research.

## Footnotes

1 The University of Winnipeg Animal Care Committee protocol#: AE04358 Institution Approval: 17 February 2015.
